# Copper Complexes as Alternative Redox Mediators in Dye-Sensitized Solar Cells

**DOI:** 10.3390/molecules26010194

**Published:** 2021-01-02

**Authors:** Alessia Colombo, Claudia Dragonetti, Dominique Roberto, Francesco Fagnani

**Affiliations:** Department of Chemistry, University of Milan, UdR-INSTM, Via C. Golgi 19, I-20133 Milan, Italy; alessia.colombo@unimi.it (A.C.); claudia.dragonetti@unimi.it (C.D.); dominique.roberto@unimi.it (D.R.)

**Keywords:** copper complexes, dye-sensitized solar cells, redox mediators

## Abstract

Thirty years ago, dye-sensitized solar cells (DSSCs) emerged as a method for harnessing the sun’s energy and converting it into electricity. Since then, a lot of work has been dedicated to improving their global photovoltaic efficiency and their eco-sustainability. Recently, various articles showed the great potential of copper complexes as a convenient and cheap alternative to the traditional ruthenium dyes. In addition, copper complexes demonstrate that they can act as redox mediators for DSSCs, thus being an answer to the problems related to the I_3_^−^/I^−^ redox couple. The aim of this review is to report on the most recent impact made by copper complexes as alternative redox mediators. The coverage, mainly from 2016 up to now, is not exhaustive, but allows us to understand the great role played by copper complexes in the design of eco-sustainable DSSCs.

## 1. Introduction

Among the world’s most important challenges for the future is to find enough supplies of clean and low-cost energy. A sustainable exploitation of solar energy would be a fascinating answer to this problem, since more energy from sunlight strikes the Earth in one hour than all the energy consumed in twelve months. In the early 1990s, dye-sensitized solar cells (DSSCs), fabricated by O’Regan and Grätzel, emerged as a realistic route for harnessing the sun’s energy and converting it into electricity [1]. Since then, a phenomenal amount of work has been dedicated to improving the photoconversion efficiency (PCE, η) of DSSCs, trying to optimize every component of the device [2,3,4,5,6,7,8,9,10,11,12,13,14,15,16,17,18,19,20,21,22].

A Grätzel-type DSSC is a device in which an electrolytic solution is contained between two electrodes made up by conductive glasses. The anode is a transparent conductive glass (ITO or FTO) presenting a layer of mesoporous TiO_2_ on which is an adsorbed dye that can be an organic molecule or a coordination complex. When visible light enters the cell through the photoanode, it interacts with the dye molecule, which is excited to a higher energetic state and transformed in its cationic form by giving an electron to the TiO_2_ layer, which transfers it to the conductive glass. By means of an external circuit, the electron flows to the cathode (also defined counter electrode), where it is given to the oxidized species of a redox couple, known as electron mediator, normally dissolved in an organic solvent such as acetonitrile. The obtained reduced form of the mediator undergoes a redox reaction in which the electron recombines with the cationic form of the dye, thus regenerating the molecule in the ground state. 

There are some fundamental parameters to provide when dealing with the performances of a DSSC and among them the most important are: the open-circuit voltage (V_oc_), representing the maximum voltage available from the solar cell, occurring when no current passes through the device; the short-circuit current density (J_sc_), which is the maximum current density present in the cell, obtained when the applied voltage is zero; the fill factor (FF), dealing with the maximum power of the cell, defined as the ratio between the maximum power of the cell and the product of V_oc_ and J_sc_, so assuming values between 0 and 1; the aforementioned photoconversion efficiency (PCE, η), representing the fraction of incident power which is converted to electric energy. 

An extensive analysis of the mentioned parameters and of other electrochemical aspects of DSSCs (also relating to copper compounds employed as redox mediators) can be found in the review published by Kavan in 2017 [23].

**Figure 1:**
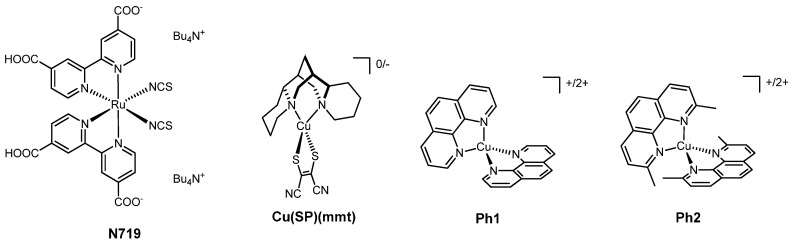
The dye N719 and the copper complexes used as redox mediators by Fukuzumi et al.

With the exception of a few organic and zinc(II) porphyrin-sensitized solar cells which reach up to 14% efficiency [20,24,25], the best performance to date in a DSSC has been achieved by using as a dye the ruthenium complex N719 (structure in Figure 1) and, as redox mediator, the couple I^−^/I_3_^−^. However, iodine-containing species present some problems for the efficient work of this type of device. Indeed, they are highly corrosive for many metals and materials. In addition, some I_2_ is always present inside the cell, and it easily escapes if the system is not properly sealed. Another problem is that I_3_^-^ competes with the dye for the absorption of light in the visible range. For these reasons, in recent years, many groups have developed DSSCs in which the redox mediators are coordination complexes, generally using as transition metals cobalt and copper.

Copper is a useful metal for energy-related applications, not only for solar cells but also in devices such as OLEDs [26,27], batteries and systems which perform the oxidation of water using electrocatalysts. The advantages of the use of copper come from many aspects: it is a relatively abundant element in the Earth’s crust, it is not as expensive as other transition metals (Ru, Pt, Pd, Ir, etc.) commonly employed, it is less toxic and it has a known coordination chemistry and redox properties (with oxidation states going from 0 to +4), and it shows important and useful photophysical and luminescence characteristics [28,29]. 

Among the difficulties arising from the use of copper complexes as redox mediators is the fact that the coordination geometry of the molecule is dependent on the oxidation state of the metal: the +1 oxidation state (corresponding to a d^10^ configuration) is generally characterized by a tetrahedral coordination, while the +2 state (d^9^ configuration) by a square planar one. This change in oxidation state when the species undergoes the redox reaction is followed by a loss of efficiency of the whole system, since a certain amount of energy is required for the reorganization of the coordination sphere of the copper cation. In order to avoid this, a good way is to have bulky substituents on the ligands in positions adjacent to the binding atoms, so that the geometry is fixed in an intermediate situation between tetrahedral and tetragonal.

Finally, in addition to the many works this review deals with in Section 2, in the years up to 2016, many examples were reported in which copper(I) compounds play not only the role of the electron shuttles but also of the sensitizers in Grätzel-type solar cells [30].

## 2. Copper Complexes as Redox Mediators in DSSCs

### 2.1. A Few Representative Examples before 2016

To our knowledge, the first use of copper complexes as redox mediators in DSSCs was reported in 2005 by Fukuzumi et al. [31]. The presented complex [Cu(SP)(mmt)] (SP = (-)-sparteine-*N,N’*; mmt = maleonitriledithiolato-*S,S’*; Figure 1) mimics the “blue copper protein” centers, with a copper atom bound to two sulfur and two nitrogen atoms, in which the blocking of the coordination geometry is provided by the protein folding. The [Cu(SP)(mmt)] complex changes its oxidation state from −1 to 0, with redox and spectral features similar to those of the “blue copper protein” centers. To carry out a comparison, the authors also employed the **Ph1** couple and the related more sterically-hindered **Ph2** (Figure 1). By using N719 as dye, **Ph1** afforded a much lower efficiency (η = 0.12%) than [Cu(SP)(mmt)] (η = 1.3%) and **Ph2** (η = 1.4%), providing evidence of the importance of bulky groups near the coordinated nitrogen atoms. As a matter of fact, while the first phenanthroline complex can easily change its coordination geometry, in the second complex, the methyl substituents are sufficiently bulky to fix the coordination in an intermediate distorted geometry. Even if the efficiency reached with **Ph2** as electron mediators was lower than that obtained with the classical I^−^/I_3_^−^ couple (η_rel, iodine_ = 51% [19], where η_rel, iodine_ represents the PCE relative to the redox mediators I^−^/I_3_^−^ set at 100%), this work played an important role and paved the way for the further development of DSSCs containing copper-based redox mediators. 

Among the various ligands which were proposed in the years up to 2016, the most used are variously substituted bipyridines, biquinolines, pyridyl-quinolines and phenanthrolines [19]. 

**Figure 2:**
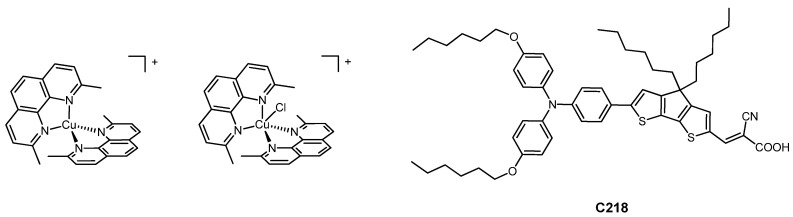
The copper-based electron shuttles and the organic dye C218 used by Wang et al.

In 2011, a record efficiency of 7% (η_rel, iodine_ = 108%) was reached by Wang et al. [32] employing [Cu(2,9-dimethyl-1,10-phenanthroline)_2_]^+^ and [Cu(2,9-dimethyl-1,10-phenanthroline)_2_Cl]^+^ as redox mediators in combination with the sterically hindered organic dye C218; the structure of both the redox mediators and the dye is shown in Figure 2.

The present review aims to present the results obtained in the field of copper-containing redox mediators in the last five years, as a further continuation of the review published in the year 2016 by Colombo, Roberto et al. [19].

### 2.2. Copper Complexes with Phenanthroline Ligands

**Figure 3:**
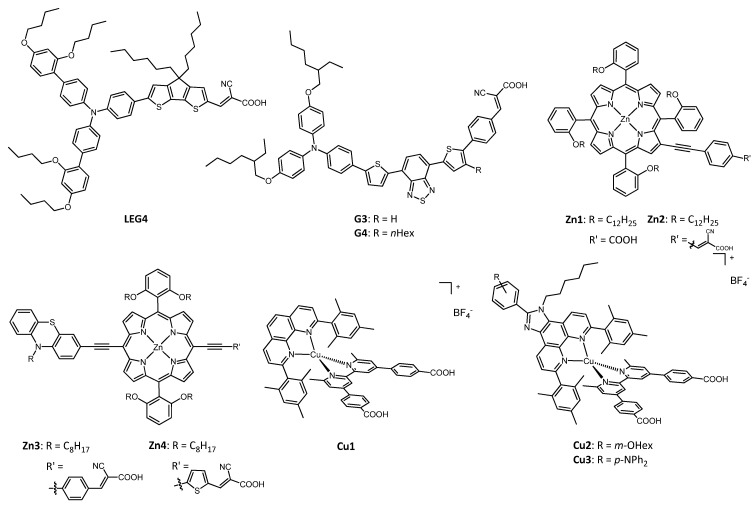
Structure of the dyes used with phenanthroline copper redox mediators.

**Figure 4:**
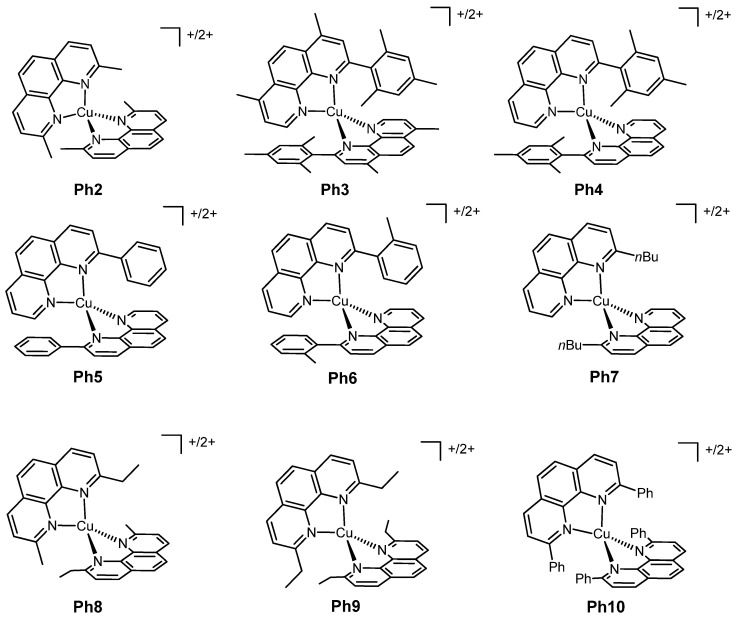
The copper complexes employed as redox mediators.

Phenanthrolines have been widely employed as ligands for Cu^+/2+^ complexes, and their use has been reported in many works in recent years. Figure 3 shows the structure of various dyes tested with the phenanthroline copper redox mediators presented in Figure 4. The photoelectrochemical data of the related solar cells are summarized in Table 1.

Freitag and coworkers published, in the year 2016, an in-depth study [33] on the copper(I)/(II) complexes **Ph2** used as redox mediators together with the organic dye LEG4 (Figure 3). They showed the advantages of this alternative with respect to the cobalt-based mediators [Co(bipy)_3_]^2+/3+^, which had a lower diffusion coefficient (6.6 10^−6^ cm^2^ s^−1^ and 9.4 10^−6^ cm^2^ s^−1^ for Co^2+^ and Co^3+^ vs 15.0 10^−6^ cm^2^ s^−1^ and 25.0 10^−6^ cm^2^ s^−1^ for Cu^+^ and Cu^2+^) and presented a slower regeneration of the LEG4 dye. The photovoltaic performance was 8.3%, with an open-circuit potential above 1.0 V under 100 mW cm^−2^ AM1.5G conditions; for the regeneration of the dye, this copper complex required a driving force of only 0.2 eV to achieve unit yield.

The authors also investigated the possible loss in efficiency of the system caused by the quenching arising from the interaction of the excited dye molecule with the Cu^2+^ complex, according to the reaction: dye* + (**Ph2**)^2+^ → dye^+^ + (**Ph2**)^+^.

This possibility was studied with steady-state emissions and Time-Correlated Single-Photon Counting (TCSPC) measurements, indicating a dynamic mechanism for the quenching, which caused a competition with the injection of electrons and thus a lower photocurrent in the device. 

The authors suggested that the overall efficiency of the DSSC could be improved by inhibiting the processes of quenching through the modification of the ligands’ structure.

In the previous year, the same group had already published a communication [34] with a study on **Ph2**; these Cu^+^/Cu^2+^ complexes were applied, together with the dye LEG4, in two different purposes: as redox mediators in the electrolytic solution and as a hole transporting material (HTM). This second option was achieved by filling the device with the normal solution of the complexes in acetonitrile (Cu^+^ 0.20 M + Cu^2+^ 0.05 M + lithium trifluoromethansulfonimide 0.1 M + *tert*-butylpyridine 0.5 M) and then allowing the solvent to evaporate, thus obtaining a solid-state DSSC (ssDSSC), often called a “zombie cell”. This solvent-free solar cell reached the best performance, with a PCE of 8.2% and a J_sc_ of 13.8 mA cm^−2^, both values being higher than the corresponding values of 6.0% and 9.4 mA cm^−2^ of the related liquid DSSC.

In order to have a reference, the authors also realized the corresponding device with the same dye and the Spiro-OMeTAD molecule as HTM, which had the lowest PCE value (5.6%) and a J_sc_ of 9.4 mA cm^−2^. 

In 2016, Magni, Colombo, Manca and coworkers published a paper in which two copper(I)/(II) electron shuttles were tested as redox couples in DSSCs, with the benzothiazole-based molecule G3 (Figure 3) playing the role of the dye [17].

The first redox couple was formed by the well-known **Ph2** complexes, while the second was represented by **Ph3** (Figure 4); these mesityl-substituted complexes had been already presented in a previous work of the same group [13] even if the cupric complex of this couple was only obtained in situ by adding NOBF_4_ to the electrolyte solution, so without isolating it. For all the four copper complexes the PF_6_^-^ was the counteranion.

In order to be stable, the cupric compound of the couple **Ph2** needed a third ligand (i.e., a Cl anion) on the Cu^2+^, while the analogous species in **Ph3** did not require this further ligand. The presence of the Cl^-^ ancillary ligand resulted in a slower regeneration of the Cu(I) complex starting from the Cu(II) one.

A proper formulation of the electrolyte solution of **Ph3** produced very promising results, with a PCE value of 4.4% under 1 sun condition, which equated the performance of the similar device containing an equimolar solution of the iodide/triiodide couple (η_rel, iodine_ = 102%). When compared with the cell sensitized with the same G3 dye but containing the **Ph2** couple, a great enhancement of the efficiency was observed (4.4% vs 1.9%); this was due to the higher ability of the cuprous species of **Ph3** of regenerating the dye molecule and, in addition, to the fact that the cupric complex of **Ph2** was less capable to rapidly regenerate the Cu(I) partner compound. 

It should be pointed out that the use of a sixfold more concentrated iodide/triiodide electrolyte solution led to a higher PCE efficiency (7.4%), but it was not possible to reach such a high electrolyte concentration with either **Ph2** or **Ph3**. Therefore, the limit of the devices produced with the aforementioned copper-based mediators was represented by their low solubility in acetonitrile, a disadvantage with respect to the highly soluble classical I^−^/I_3_^−^ redox couple.

The great potential of phenanthroline copper complexes as redox mediators was furtherly shown in the work of Pizzotti, Roberto, Caramori et al. of 2017 [35], in which two Zn^2+^ tetraaryl-substituted porphyrin dyes (Figure 3) were tested together with the three Cu^+/2+^ couples, **Ph2**, **Ph3** and **Ph4** (Figure 4). 

Concerning the dye, the improvement in the device efficiency came from the introduction in the porphyrinic system of long *ortho*-dodecyloxy chains, which hindered the aggregation of the dye molecules on the surface of the titanium dioxide layer, thus increasing the electron injection on the photoanode. The anchoring group was a carboxylic (Zn1) or cyanoacrilic (Zn2) moiety, connected to the porphyrinic core through an ethynylphenyl spacer. 

The first dye, Zn1, was coupled with five different redox mediators: the three aforementioned copper-phenanthrolines couples, the traditional I^-^/I_3_^-^ and the promising cobalt-based complexes [Co(dtb-bipy)_3_]^2+/3+^ (dtb-bipy = 4,4′-di-*tert*-butyl-2,2′-bipyridine). The **Ph4** complexes gave the best results and led to a considerable 75% increase in PCE (3.7% vs 2.1%) when compared with the reference couple **Ph2**; this value was also the best when compared with iodine- and cobalt-based electron shuttles (PCE = 3.4% and 2.9%, respectively), giving an excellent η_rel, iodine_ of 109%.

In addition, comparison with the cells sensitized with the **Ph3** mediators (η = 2.9%, η_rel, iodine_ = 85.3%) shows that removal of the two methyl groups on positions 4 and 7of the phenanthroline system was a way to improve the efficiency of the DSSC.

The second dye, Zn2, was tested with the best performing redox mediators, i.e., **Ph4**, but with worse results with respect to the Zn1 porphyrin (J_sc_ = 4.8 mA cm^−2^ and PCE = 2.7%), suggesting that the simple carboxylic anchoring group is more effective than the cyanoacrilic one.

This work represents the first example of the combination of Zn-based porphyrin sensitizers with efficient Cu^+^/Cu^2+^ redox mediators, opening the way for further investigations aiming at low cost production and large-scale diffusion of DSSC devices.

A very recent application of copper-based electron shuttles together with Zn^2+^ porphyrin dyes is in the work of Imahori and coworkers of 2020 [36], in which the authors coupled the aforementioned redox couple **Ph2** and the bipyridine-based complexes **BP2** (structure in Figure 6) with the two new push–pull porphyrin sensitizers, Zn3 and Zn4 (Figure 3). The aim of this new molecular structures was to enhance the photovoltaic properties of the dye by means of the proper selection of constituents in the porphyrinic system.

The most promising efficiency values were achieved by the combination of the dyes with the **BP2** couple (PCE = 5.07% for Zn3 and 4.36% for Zn4). The bipyridine-based redox mediators also provided slightly higher open-circuit voltages and fill factors; moreover, according to the authors, the remarkable difference in the efficiency mainly came from the short-circuit current density (J_sc_).

To date, the η value exhibited by the combination of Zn3 and **BP2** in this work represents the highest result in the field of Cu^+^/^2+^ redox mediators used together with porphyrin-based DSSCs. 

In a 2018 paper of Magni, Colombo, Manca and coworkers [37], many different phenanthrolines substituted in position 2 were tested as ligands for copper-based redox mediators in DSSCs. The devices presented the organic dyes G3 and G4 and in all cases the copper complexes had the PF_6_^−^ anion. The authors employed different substituents, both aromatic rings (phenyl, mesityl, *o*-tolyl) and alkyl chains (*n*-butyl), with the aim of fixing the geometry by virtue of the steric hindrance provided by the introduced moieties. The studied copper couples were **Ph4**, **Ph5**, **Ph6** and **Ph7** (structure in Figure 4).

The test on the obtained copper-based couples involved the preparation of electrolyte solutions containing a typical 10:1 concentration ratio between the Cu^+^ complex (0.15 M) and the Cu^2+^ species (0.015 M); moreover, lithium trifluoromethansulfonate (LiOTf) 0.1 M and *tert*-butylpyridine (TBP) 0.25 M were used as additives. In order to have a reference, electrolytic solutions with I^−^/I_3_^−^ and [Co(bipy)_3_]^2+/3+^ were prepared in identical conditions, and in all cases, the employed dyes were G3 and G4 (Figure 3).

In these experiments, the Cu-based couples **Ph4** and **Ph6** appeared to be very efficient and showed better results when compared with the reference redox mediators. As a matter of fact, by using G3 as the dye, they had a PCE of 5.6% and 6.0%, respectively, thus overcoming the efficiency reached by both the cobalt- and iodine-based mediators (4.7% and 5.2% respectively), with a remarkable improvement and a η_rel, iodine_ of 107% for **Ph4** and 115% for **Ph6**. Similar PCE efficiencies were reached with G4.

Additionally, the couples, **Ph5** and **Ph7**, afforded very promising results, especially in the case of the combination of **Ph7** with G3, with an outstanding photon-to-current conversion efficiency of 5.7% and a η_rel, iodine_ of 109%.

A similar approach was presented by Imahori et al. in their 2020 work [38], with the difference that the redox couples were tested together with the organic dye LEG4 and that the phenanthrolines had substituents also in position 9. The proposed copper-containing complexes were **Ph7**, **Ph8**, **Ph9** and **Ph10**, whose structure can be seen in Figure 4.

The cuprous species had TFSI^−^ (bis(trifluoromethane)sulfonimide) as anion, while the cupric ones both TFSI^−^ and Cl^−^.

To evaluate the performances of these new complexes, the authors realized a DSSC with the already known couple **Ph2**, which still exhibited the highest efficiency (6.29%), since this couple provided the highest open-circuit voltage (1.03 V) and a high value of the short-circuit current density (10.0 mA cm^−2^). 

A close efficiency of 5.53% was given by the complexes **Ph7**, having a very similar J_sc_ (10.6 mA cm^−2^) but a smaller V_oc_ (0.842 V). Even if the other three couples had a similar voltage with respect to the *n*Bu-substituted phenanthroline complexes, the lower J_sc_ values limited the overall efficiency of the corresponding devices, thus leading to worse results. 

An important step towards environmentally friendly DSSCs was made in 2018 by Colombo et al. by means of the realization of the first example of a “full-copper” device, which contained a copper-based dye coupled with redox mediators made up of copper(I)/(II) complexes [39].

The redox couples used in this work were the aforementioned **Ph2** and **Ph7** (Figure 4), as hexafluorophosphate salts. The dye (Cu1, Figure 3) was a relatively simple heteroleptic complex of Cu(I) having two bidentate ligands which were different since they played different roles: the former was a 2,9-dimesityl-1,10-phenanthroline (dMesPhen) which acted as a light-absorbing moiety for the whole complex, while the latter was a dimethyl-substituted 2,2′-bipyridine bearing phenyl spacers and two anchoring carboxylic groups. The mesityl groups in the positions adjacent to the nitrogen atoms not only fixed the coordination geometry in a distorted tetragonal system, preventing an energetic loss associated with the change from Cu^+^ to Cu^2+^, but also hampered the formation of the two corresponding homoleptic complexes starting from the heteroleptic ones, since the in situ formation of Cu(dMesPhen)_2_ was made not possible by the remarkable steric hindrance of the ligands. 

As above reported, among the problems faced when dealing with copper-based redox couples is the limited solubility of the cupric species in organic solvents such as the commonly used acetonitrile: here, the maximum amount of Cu^2+^ complex was around 0.017 M. Under these conditions (0.17 M Cu(I) and 0.017 M Cu(II) in the presence of 0.1 M LiTFSI), the coupling of **Ph2** and **Ph7** with the dye Cu1 gave efficiency values of 1.3% and 2.0%, respectively; the PCE was lower than those obtained with the same concentrations of electrolytic solution of I^−^/I_3_^−^ (3.6%), and consequently, the η_rel, iodine_ was 36.1% for the dimethyl-substituted redox couple and 55.5.% for the other one. It was observed that an increase in the solubility of the copper(II) complex would allow a higher concentration of the redox mediator and could be essential to achieve an improvement in the efficiency of copper-based DSSCs. In any case, this work showed how copper electron shuttles can be combined with copper dyes to give performing low-cost solar cells.

The following year, the same group published another paper on the implementation of full-copper DSSCs [40]. The main purpose was the development of another two heteroleptic copper(I) complexes which could find application as dyes when coupled with the already presented redox mediators **Ph2** and **Ph7**. These new complexes were different from the previously studied Cu1, since they had an extended aromatic system on the mesityl-substituted phenanthroline ligand: the central ring of the molecule was condensed with an imidazole, which presented a phenyl moiety bearing a *m*-OHex group in the first case (Cu2) and a *p*-NPh_2_ moiety in the other (Cu3). The structure of the two new dyes is in Figure 3. This change aimed to enhance the absorption of solar light; moreover, one nitrogen atom of the imidazole was alkylated with an hexyl chain, to improve the solubility of the dye, to prevent an easy deprotonation of the NH group and the aggregation of the dye molecules onto the TiO_2_ surface, and also to protect the surface of the semi-conductor, thus avoiding the recombination of the dye^+^ species with the electron previously given to the photoanode.

The two new copper dyes were tested with the two copper-based redox couples, **Ph2** and **Ph7**, and also with the common iodide/triiodide electrolytes. 

It turned out that the new dyes with these ancillary phenanthrolines did not show better efficiencies when compared to Cu1: the involvement of the π-delocalized phenanthroline in the excited state, originating visible light absorption, became detrimental due to a reduction in the charge transfer directionality. The best results were observed using the couple **Ph7**, which gave PCE values of 1.4% when coupled with Cu1, 1.2% with Cu2 and 1.0% with Cu3, reaching a η_rel, iodine_ of 22.9%, 21.4% and 17.2%, respectively (η_iodine_ = 3.05%).

Moreover, other devices were prepared employing the same copper-based dyes, electron shuttles and additives, but with the additional presence of a 0.05 M solution of guanidinium iodide. In this case, the efficiency values were only slightly better than the previous ones.

It turned out that the overall performances of the DSSCs filled with the copper-containing redox mediators were mainly hampered by an important decrease in both the J_sc_ and the FF.

In 2019, the same authors provided evidence on the crucial role of an additive such as 4-*tert*-butylpyridine (TBP) in the performance of these sustainable “full-copper” DSSCs [41]. Thanks to this additive and to the proper concentration of copper species, a 2.51% PCE efficiency and a η_rel, iodine_ of 82.3% (82.8% in the presence of guanidinium iodide) could be reached upon combination of the dye Cu1 with **Ph7** as redox mediators. 

**Figure 5:**
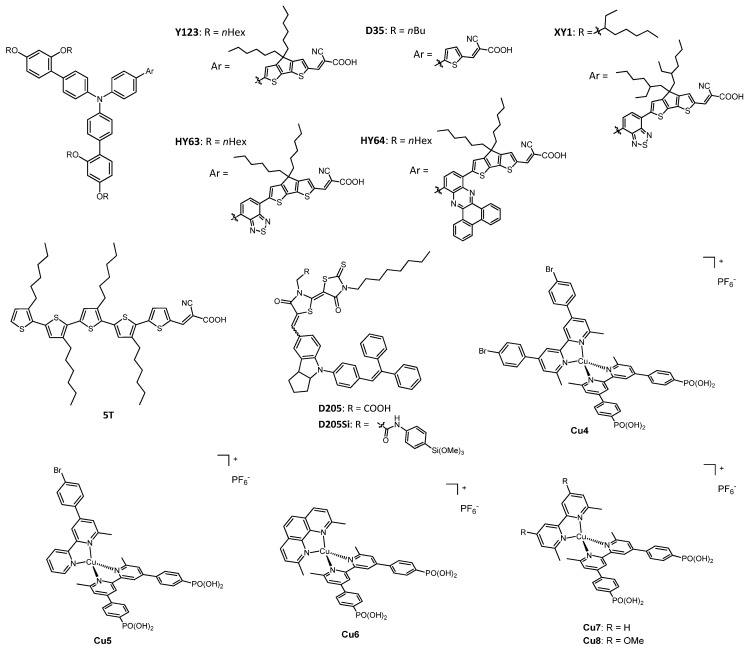
Structure of the dyes used with bipyridine copper-based redox mediators.

**Figure 6:**
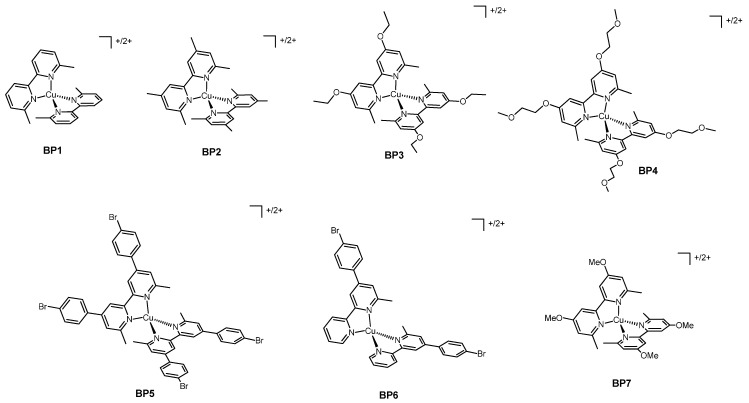
The bipyridine copper complexes employed as redox mediators.

The effect of TBP on the mass-transport and on the charge transfer kinetic at the counterelectrode was the subject of an in-depth study carried out by Kavan and coworkers in 2016 [42]. Normally, the counterelectrodes in DSSCs present, as catalysts, a layer of platinum or of poly(3,4-ethylendioxythiophene) (known as PEDOT), but in this work, the authors explored the possibility of employing graphene. All the aforementioned three catalysts were tested with the dye Y123 (Figure 5) and with the redox mediators **Ph2** (Figure 4), **BP1** and **BP2** (Figure 6). This first use of graphene gave very promising results, reaching a comparable activity with respect to PEDOT and also outperforming the activity of platinum. The usefulness of TBP was due to its ability to slower the charge-transfer kinetic and the diffusion rate in the case of copper-based redox mediators. This effect was not present for Co^2+/3+^ redox couples, since their coordination sphere was less sensitive to the presence of TBP in the electrolytic formulation.

In 2017, the same group published an article [43] in which they presented a novel type of cathode catalyst for DSSCs. This new catalyst was a multicomponent material made of Pt, PtO_x_, graphene oxide and stacked graphene platelet nanofibers; when coupled with the dye Y123 and the redox mediators **BP2**, it appeared to be much more effective than the widely-used Pt and PEDOT.

The importance of considering the proper base to be added to the electrolytic solution had also already been shown in the work of Hagfeldt, Kavan et al. from 2018 [44]. In this work, the authors tested the common dye Y123 and the redox couple **BP2** with four different pyridine-based additives: in addition to the well-known TBP, they evaluated the effect of 2,6-di-*tert*-butylpyridine, 4-methoxypyridine and 4-(5-nonyl)pyridine on the electrochemical properties of the prepared DSSCs. While these molecules did have an effect the Cu(I)/(II) electron shuttles, a change in the properties of the control device filled with the [Co(bipy)_3_]^2+/3+^ couple was not observed.

### 2.3. Copper Complexes with Bipyridine Ligands

Another widely used ligand for copper is 2,2′-bipyridine (generally referred to as bipy), and complexes which employ this molecule have often been tested as new redox mediators for DSSCs.

Figure 5 shows the structure of various dyes tested with the bipyridine copper redox mediators presented in Figure 6. The photoelectrochemical data of the related solar cells are summarized in Table 2.

In 2016, Freitag and coworkers studied two Cu^+/2+^ couples as possible redox mediators [45]: starting from the already known complexes **Ph2**, whose geometry was fixed by the methyl groups in the positions 2 and 9 of the phenanthroline, the first investigated couple was **BP1**, with a structure very close to that of **Ph2**. The second bipyridine couple was **BP2**, with two more methyl substituents on the aromatic rings. Both the change from phenanthroline to bipyridine and the introduction of the additional methyl groups in positions 4 and 4′ aimed to tune the redox properties of the studied couples. All the presented copper complexes had the TFSI^-^ anion and were used together with the organic dye Y123, whose structure (Figure 5) was similar to that of LEG4 with the only difference of having *n*-hexyloxy chains on the phenyl rings instead of the *n*-butyloxy ones. 

The usefulness of these mediators came from their ability to rapidly regenerate the dye in the fundamental state by giving an electron to the cationic form from the cupric complex, preventing the back-transfer between the titania surface and the oxidized sensitizer. To achieve high photovoltage values, the reduction in the dye^+^ species should occur with a low driving force.

At full sunlight illumination, the V_oc_ for all the three couples, **Ph2**, **BP1** and **BP2**, was above 1.0 V. The highest J_sc_ was reached in the case of **BP2**, while all DSSCs containing the mentioned electron shuttles had a power conversion efficiency higher than 10.0%: PCE = 10.3% for both **Ph2** and **BP2** and 10.0% for **BP1**.

Since these copper complexes showed good results even in 0.2 sun conditions, the authors stated that they could find a suitable employment in indoor-focused devices.

In addition to the mentioned work of Freitag et al., the potential usefulness of **BP2** complexes as redox mediators is shown by the many papers which dealt with them in recent years.

Thus, a further study on **BP2** was published by Grätzel, Hagfeldt et al., in 2017 [46]. In this case, the redox mediators were coupled with different organic dyes specifically designed for this purpose, namely, the molecules D35 and XY1: the former dye presented a Donor-π-Acceptor (D-π-A) structure, while the latter a D-A-π-A one (Figure 5). The co-sensitization of the dyes allowed for an increase in the short-circuit photocurrent, since the absorption of the D35 dye was in the blue and green regions of visible light, while the XY1 absorbed yellow and red light; in this way, the light-harvesting ability of the DSSC was remarkably widened, ranging from 400 to 650 nm.

According to the authors, the combination of the two sensitizers led to useful synergistic interactions between the dye species, with one compound able to hamper the unwanted interactions of the other with the titania and also with itself (in the case of dye aggregation). Furthermore, the coupling of these dyes with the copper-based redox mediators prevented a fast unfavorable back-electron transfer, thanks to the tightly packed layer of dye molecules on the semiconductor surface. 

This blended device reached a PCE of 11.3% (the highest value at the time of the article publication) with a D35:XY1 ratio of 4:1, with whom values of Jsc = 16.2 mA cm^−2^ and FF = 68% were associated. In all the tested co-sensitization ratios, the V_oc_ was above 1.0 V, with a maximum value of 1.1 V for pure D35.

A further test concerned the indoor-light conditions under which these devices were tested: while in the case of solar emission and outdoor conditions, one employs the irradiance (in W/m^2^) as the unit to describe a light source, for indoor environments, the commonly used unit is the illuminance (in lux), which connects the intensity to the human perception. To make a comparison, a typical indoor light of 1000 lux corresponds to only ~1% of the standard 100 mW cm^−2^ solar source.

Under 1000 lux intensity, a PCE value of 28.9% was reached for the **BP2** solution in acetonitrile, with a power output (P_out_) of 88.5 μW cm^−2^, which outperforms a classical reference Ga-As solar cell. Promising values were obtained also in the case of a 200 lux source (PCE = 25.5% and P_out_ = 15.6 μW cm^−2^) and with the use of propionitrile as solvent (efficiencies of 27.4% at 1000 lux and of 22.3% at 200 lux).

In 2020, the same **BP2** couples and the dye XY1 were tested in the paper of Freitag, Robertson et al. [47], where the authors realized a co-sensitized DSSC with the aim of reducing the production costs of the devices while maintaining the performances already shown in their previous work. The XY1 dye was coupled with the less expensive dye 5T (Figure 5), which presented a π-A structure.

The main problem faced with the dye XY1 concerned its synthesis: it involved 12 steps in total, with three of them requiring the use of the Pd(PPh_3_)_4_ catalyst. The use of this dye alone was not optimal, since the cost of its production exceeded that of the traditional ruthenium-based dye N719, making it not useful from an economic point of view. 

Consequently, the molecule 5T was chosen as a proper co-sensitizer considering some advantages: the UV/Vis absorption peaks were complementary to those of XY1, and the synthesis was both cheaper and easier. The DSSCs containing the two sensitizers reached an average PCE of 9.1% at 1 sun and 9.4% at 0.1 sun.

The cost analysis carried out by the authors showed that blending the XY1 and 5T dyes provided a reduction in the costs (evaluated on the basis of the costs of the syntheses and of the starting materials) of about 70%, with results similar to those of the DSSCs containing only XY1.

This work represents an important analysis of some aspects which are often neglected when dealing with the design of efficient dyes, i.e., the usefulness of co-sensitization and the evaluation of costs, to achieve a real possibility of commercialization of this kind of devices.

In the same year, Zhu, Grätzel and coworkers coupled the **BP2** complexes with two new organic dyes [48]. The structure of these new molecules was a derivative of the well-known dye Y123 previously mentioned, which was among the most efficient and widely used sensitizers for copper-based DSSCs. In this case, the authors aimed to extend the spectral response towards lower energies by means of the introduction of two different electron-withdrawing spacers in the D-A-π-A molecular structure: a benzothiazole (BT) for HY63 and a phenanthrene-fused-quinoxaline (PFQ) for HY64 (Figure 5). 

Thanks to this modification, they succeeded in red-shifting the absorption by about 50 nm with respect to the dye Y123. 

It became evident from the various experiments that the PFQ moiety was a useful building block for the proper design of long-wavelength-responsive dyes for DSSCs. In fact, the PFQ component of the HT64 molecule was widely compatible with copper-based electron shuttles, since the association with the **BP2** couple was made difficult by the large and rigid structure of the moiety, which reduced the possibility of interaction of cupric species with the electrons in the conduction band of the titanium dioxide.

Moreover, while the HY63 dye required the presence of the chenodeoxycholic acid as co-adsorbent in order to achieve the best values of efficiency (10.3%), it was not needed for HY64, whose PCE and J_sc_ parameters reached a value of 12.5% and 15.76 mA cm^−2^, respectively. 

To date, this represents the best value of a DSSC containing a single dye and a copper-based redox couple.

The authors concluded that the PFQ-containing dye HY64 should be preferred to the BT-based one, which was not as efficient as the former compound in preventing interfacial charge recombinations.

The widely used **BP2** redox mediators were coupled, in 2020, by Glinka, Ziółek et al. with other organic dyes, such as the indoline-based sensitizers D205 and D205Si [49]; the latter compound presented a silyl-anchoring group instead of the carboxylic moiety of the former one (Figure 5). 

Both dyes were studied together with the traditional redox mediators I^−^/I_3_^−^ and with the aforementioned **BP2**; the results were later compared with those collected for the devices employing the [Co(bipy)_3_]^2+/3+^ couple.

The authors chose the triphenylamine dye Y123 as a reference and then studied the possibility of blocking the recombination phenomena (which can occur on the interface semiconductor/dye/electrolyte) through the use of cucurbit [7] uril (CB7) molecules. The CB7 molecule is a macrocyclic compound formed by glycouril oligomers, arranged in such a way that they form a cage similar to those of cyclodextrins. The molecule with 7 repeating units was selected because of its good solubility in water and on the basis of its size, suitable to contain organic molecules.

A further way to hamper the electron recombination and to avoid the desorption of the dye from the semiconductor was to apply a multicapping (MC) procedure: many different carboxylic, sulfonic or phosphonic acids with different steric hindrance and pK_a_ values were supposed to be adsorbed on the TiO_2_ surface in the voids between the dye molecules. However, it was not possible to completely prevent the dye desorption, even if it was remarkably reduced.

The consequences of the CB7 and MC interface modifications depended on the considered dye: the Y123-sensitized reference cells underwent a decrease in electron lifetime, resulting in a lower V_oc_ of 1.03 V for MC and 1.01 V for CB7, starting from a value of 1.07 V for the pristine cell.

Nevertheless, the use of these techniques was beneficial for the cells containing the dyes D205 and D205Si: an increase in J_sc_ was observed and, in particular for the alkoxysilyl derivative, whose PCE achieved values of 4.37% and 4.41% for MC and CB7, respectively, with a great relative rise with respect to the original 3.92%.

In addition to the aforementioned pioneering work of Freitag and coworkers of 2015 [34], described in the “copper-based redox mediators with phenanthroline ligands” section, “zombie” DSSCs were described also in 2020, in a paper of Hagfeldt and coworkers [50]. In this work, the widely used organic dye Y123 was tested with two new copper-based redox couples, which maintained the core structure of the well-known 6,6′-dimethyl-2,2′-bipyridine (to fix the geometry), but with different substituents in the positions 4 and 4′. One problem faced while realizing a zombie cell was the fact that the slow evaporation of the organic solvent could lead to the crystallization of the copper complexes, which reduced the performances of the device. In order to decrease the possibility of lattice packing and hence the crystallization, it appeared useful to introduce aliphatic chains on the ligands. The selected substituents were the ethoxy group and the 2-methoxyethoxy chain, thus obtaining the couples **BP3** and **BP4** (Figure 6). For comparison, the already known couple **BP2** was employed to realize control cells.

X-ray diffraction measurements on single crystal of **BP3** showed that the coordination geometries of these species were very similar to those of the reference compounds **BP2**, suggesting the absence of negative effects of the ligands on the structure around the copper cation. Additionally, **BP4** were the least crystalline among the considered redox mediators.

Using these three copper-based redox mediators, both traditional liquid and solid state DSSCs were prepared; the liquid devices presented good performances, with a PCE value of 10.4% and 10.2% for **BP3** and **BP4**, respectively. On the other hand, the features of the zombie cells were very poor (with an efficiency of 5.43% for **BP3** and 2.36% for **BP4**), due to many problems such as the strong hysteresis and the remarkable charge transfer resistance caused by the ligands. In sum, the modifications on the bipyridine ligands (with respect to the popular **BP2** couple) did not involve different features on the liquid DSSCs, but had a great impact on the zombie cells.

As mentioned in Section 2.2 (copper complexes with phenanthroline ligands), copper electron shuttles can be combined with copper dyes to produce performing low-cost solar cells. In 2018, Housecroft et al. [51] investigated the performances of DSSCs based on heteroleptic copper(I) sensitizers combined with homoleptic Cu^+^/Cu^2+^ redox mediators.

All the dye complexes (Cu4-Cu8, Figure 5) presented as an anchoring ligand a bipyridine bearing a phosphonic acid moiety and as ancillary ligand a differently substituted bipyridine or phenanthroline. The redox mediators (**BP1**, **Ph2**, **BP5**-**BP7**; structure in Figure 6) were homoleptic copper compounds whose ligand was among the previously mentioned ancillary ligand of the dyes; the Cu(II) species was obtained from the corresponding Cu(I) molecule by firstly oxidating it using NOBF_4_ and then exchanging the anion to have the PF_6_^-^ salt.

The electrolytic solution of the produced DSSCs contained a 5:1 ratio of the cuprous/cupric complexes, with different amounts (concentration of Cu(I) ranging from 0.02 M to 0.20 M) of the compounds depending on their solubility in acetonitrile; as additives, 0.5 M TBP and 0.1 M LiPF_6_ were present.

Very good photoconversion efficiency (2.06%) and short-circuit photocurrent (4.01 mA cm^−2^) were obtained by coupling the dye Cu8 together with the redox couple **BP7**.

This work confirms the great potential of full-copper DSSCs.

### 2.4. Copper Complexes with Other Types of Ligands

**Figure 7:**
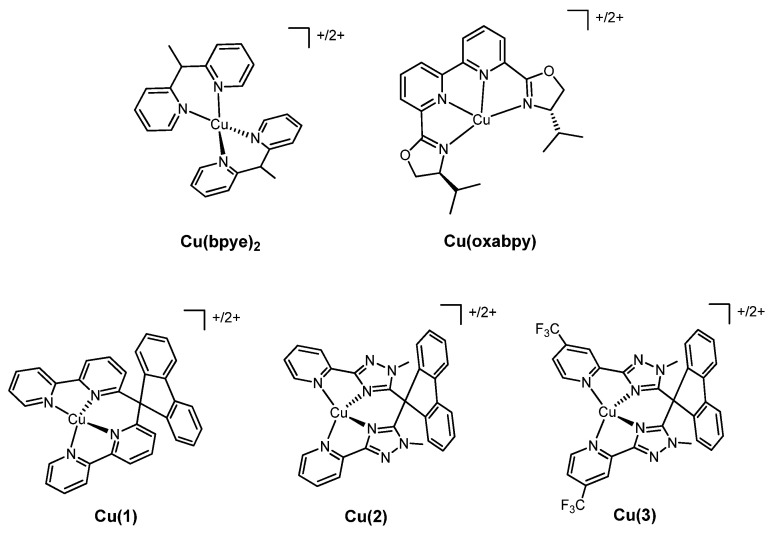
Other copper complexes employed as redox mediators.

In addition to the common phenanthrolines and bipyridines, other molecules have been tested as promising ligands for copper(I)/(II) complexes to be employed as redox mediators in DSSCs. Their structure is reported in Figure 7, whereas the photoelectrochemical data of the related solar cells are shown in Table 3.

A first example was given by Kloo and coworkers in 2016 [52]. In their work, the authors synthesized and studied the complexes **Cu(bpye)_2_** (where bpye = 1,1-bis(2-pyridyl)ethane, see Figure 7) coupling them with the well-known organic dye LEG4 (Figure 3). As a comparison, they also set up a DSSC filled with the couple [Co(bipy)_3_]^2+/3+^, preparing the electrolytic solution in identical formulation, with the same number of reduced/oxidized species and of both the additives LiClO_4_ and 4-*tert*-butyl-pyridine.

In all the experiments, the copper-based redox mediators provided better results with respect to the cobalt-containing ones, with a short-circuit current density of 14.1 mA cm^-2^ and a PCE of 9.0% at 1 sun; these remarkable values came from fast electron self-exchange reactions, proper redox potentials and a rapid regeneration of the sensitizer.

Another approach towards the fixation of the coordination geometry of copper cations, in order to reduce the energetic loss which follows the change in oxidation state, was presented in the 2018 work of Freitag et al. [53]. In that paper, the ligand was the tetradentate molecule 6,6′-bis(4-(S)-isopropyl-2-oxazolinyl)-2,2′-bipyridine (oxabpy), binding the copper atom through the four nitrogen atoms. The **Cu(oxabpy)** complexes (Figure 7) were coupled with the organic dye Y123 (structure in Figure 5).

The use of liquid electrolytes is among the main problems associated with the production of DSSCs, since the organic solvent, in which the electron shuttles are dissolved, can easily evaporate and escape from the device if it is not properly sealed. Considering the opposite cases of the “traditional” cell with the liquid electrolyte and the “zombie” cell with a solid hole transport material, the device presented in this paper showed intermediate features, since the particular redox couples allowed for a high viscosity of the solution, more than 2.5 times the corresponding reference solution containing the well-known **BP2** complexes.

The solar cells filled with the **BP2** couple still had a higher open circuit voltage (1040 mV) when compared with the device containing the new **Cu(oxabpy)** mediators (920 mV), but this result could be regarded as not completely negative, considering that the voltage difference was of only 120 mV, whereas the formal redox potential difference was of 210 mV.

Additionally, the J_sc_ and the PCE were lower for the tetradentate ligand, with values of 9.75 mA cm^-2^ and 6.2% versus 10.5 mA cm^−2^ and 7.8%.

Another three tetradentate rigid ligands (Figure 7) for copper were tested in 2020 by Fortenberry, Delcamp, Jurss and coworkers [54]. The biphenyl moiety of these molecules forced the four nitrogen atoms in a pseudo-tetrahedral arrangement which allowed the chelation of the metal center while preventing the change towards the square planar coordination geometry. The new redox mediators were coupled with the organic dye Y123 and with the two different counterelectrodes PEDOT and Pt. Moreover, the aforementioned complexes **Cu(bpye)_2_** were used as a benchmark for the comparison of the results. The tunability of the features of the ligands was studied by varying the dentate substituents on the rigid biphenyl structural unit: in the complexes **Cu(1)** the molecule bound the copper cation with four pyridyl rings, while in the **Cu(2)** couple through two pyridyil-methyltriazole moieties. Finally, a further variation was represented by the introduction of the electron-withdrawing group –CF_3_ on the pyridine rings of the ligand **2**, thus providing the complexes **Cu(3)**.

The use of **Cu(2)** together with a platinum counterelectrode gave a value of J_sc_ very close to that of [Co(bpy)_3_]^2+/3+^-Pt (14.1 mA cm^−2^ vs 14.2 mA cm^−2^) and higher than that of **Cu(bpye)_2_**-Pt (9.7 mA cm^−2^). In order to explore the stability over time of these redox mediators, the couples **Cu(1)** and **Cu(2)** were tested over a wide period of time, and the authors found that the highest stability was obtained with the device based on **Cu(2)**-Pt. Both the stability over time and the better value of J_sc_ for the mediators **Cu(2)** came from its rigid tetradentate ligand, which efficiently reduced structural changes upon redox cycles.

## 3. Conclusions

Although redox mediators constitute an essential component of a dye-sensitized solar cell, much less research effort has been devoted to them compared to dyes. For many years, the traditional redox mediator has been the iodide/triiodide couple which has some disadvantages such as the volatility of I_2_ complicating long-term cell sealing, corrosion problems, large required driving force for dye regeneration. In recent years, the use of copper complexes as redox mediators appeared to be a solution to the problems of the iodide/triiodide couple, giving a new impulse to dye-sensitized solar cells research. Copper is a relatively abundant element in the Earth’s crust, and well-designed copper complexes demonstrate to be excellent redox mediators with suitable redox potentials, good dye regeneration properties with small driving force requirement.

In the last 5 years, various DSSCs using copper complexes as redox mediators reached outstanding photon-to-current conversion efficiencies, sometimes equating or even surpassing that obtained with the classical iodide/triiodide couple. In several investigations, PCE values exceeding 10% were reached by combining a bipyridine copper complex with a suitable dye. Excellent performance was obtained not only for the typical case of the complex dissolved in a liquid electrolyte but also for a solid-state solar cell obtained after electrolyte evaporation. Because copper-based redox mediators can show good results even at very low lighting conditions, they could find a suitable application both in outdoor and indoor (with artificial light of low intensity) devices. Remarkably, they can be combined to copper dyes to give performing low-cost full-copper solar cells.

Copper complexes are excellent alternative redox mediators which will surely allow even more efficient eco-sustainable low-cost dye-sensitized solar cells to be obtained in the near future.

## Figures and Tables

**Table 1:** Photoelectrochemical data of solar cells filled with phenantroline copper complexes as redox mediators. ^1^

Dye	Redox Couple	Voc (mV)	Jsc (mA cm^−2^)	FF	PCE (%)	η_rel, iodine_ (%)	CE ^2,3^	Ref
LEG4	Ph2 ^4^	1020	12.6	0.62	8.3	-	PEDOT	[33]
LEG4	Ph2 ^5^	1035	9.4	0.62	6.0	-	PEDOT	[34]
LEG4	Ph2 ^6^	1010	13.8	0.59	8.2	-	PEDOT	[34]
LEG4	Ph2 ^7^	1030	10.1	0.592	6.12	-	Pt	[38]
LEG4	Ph7 ^7^	836	10.4	0.634	5.53	-	Pt	[38]
LEG4	Ph8 ^7^	794	7.82	0.505	3.09	-	Pt	[38]
LEG4	Ph9 ^7^	803	6.45	0.449	2.33	-	Pt	[38]
LEG4	Ph10 ^7^	799	4.30	0.602	2.06	-	Pt	[38]
G3 ^8^	Ph2 ^9^	860	3.8	0.59	1.9	44.2 ^10^	Pt	[17]
G3 ^8^	Ph3 ^9^	720	9.3	0.66	4.4	102 ^10^	Pt	[17]
G3 ^8^	Ph3 ^9^	740	8.2	0.67	4.1	95.3 ^10^	PEDOT	[17]
G3 ^8^	Ph4 ^11^	830	11.4	0.59	5.6	107 ^12^	Pt	[37]
G3 ^8^	Ph5 ^11^	880	8.0	0.69	4.9	94.2 ^12^	Pt	[37]
G3 ^8^	Ph6 ^11^	870	11.1	0.62	6.0	115 ^12^	Pt	[37]
G3 ^8^	Ph7 ^11^	860	10.1	0.66	5.7	109 ^12^	Pt	[37]
G4 ^13^	Ph4 ^11^	840	11.7	0.54	5.3	-	Pt	[37]
G4 ^13^	Ph5 ^11^	810	10.2	0.58	4.8	-	Pt	[37]
G4 ^13^	Ph6 ^11^	870	11.1	0.62	6.0	-	Pt	[37]
G4 ^13^	Ph7 ^11^	780	10.1	0.63	4.9	-	Pt	[37]
Zn1	Ph2 ^14^	860	3.5	0.70	2.1	61.7 ^15^	PEDOT	[35]
Zn1	Ph3 ^14^	680	5.6	0.77	2.9	85.3 ^15^	PEDOT	[35]
Zn1	Ph4 ^14^	810	5.9	0.77	3.7	109 ^15^	PEDOT	[35]
Zn2	Ph4 ^14^	750	4.8	0.74	2.7	-	PEDOT	[35]
Zn3 ^16^	Ph2 ^17^	890	6.89	0.65	3.98	-	Pt	[36]
Zn3 ^16^	BP2 ^17^	907	8.33	0.67	5.07	-	Pt	[36]
Zn4 ^16^	Ph2 ^17^	864	5.06	0.69	3.03	-	Pt	[36]
Zn4 ^16^	BP2 ^17^	892	6.86	0.71	4.36	-	Pt	[36]
Cu1	Ph2 ^16^	750	4.7	0.36	1.3	36.1 ^17^	Pt	[39]
Cu1	Ph7 ^16^	610	6.3	0.53	2.0	55.5 ^17^	Pt	[39]
Cu1	Ph2 ^16^	694	2.9	0.36	0.7	11.5 (14.9) ^18^	Pt	[40]
Cu1	Ph7 ^16^	593	3.8	0.61	1.4	22.9 (29.8) ^18^	Pt	[40]
Cu1	Ph7 ^19^	622	5.77	0.70	2.51	82.3 (82.8) ^20^	Pt	[41]
Cu2	Ph2 ^16^	647	2.6	0.35	0.6	10.7 (11.5) ^18^	Pt	[40]
Cu2	Ph7 ^16^	570	3.2	0.67	1.2	21.4 (23.1) ^18^	Pt	[40]
Cu3	Ph2 ^16^	370	0.9	0.37	0.1	1.7 (1.9) ^18^	Pt	[40]
Cu3	Ph7 ^16^	573	2.8	0.62	1.0	17.2 (17.8) ^18^	Pt	[40]

^1^ AM 1.5 simulated light source; input intensity of 100 mW cm^−2^. ^2^ CE: counterelectrode. ^3^ PEDOT: poly(3,4-ethylendioxythiophene). ^4^ 0.20 M Cu(I) + 0.04 M Cu(II) + 0.1 M LiTFSI + 0.5 M TBP. ^5^ 0.20 M Cu(I) + 0.05 M Cu(II) + 0.1 M LiTFSI + 0.5 M TBP. ^6^ as HTM in the zombie cell. ^7^ 0.2 M Cu(I) + 0.05 M Cu(II) + 0.1 M LiTFSI + 0.5 M TBP. ^8^ 0.2 mM G3 + 30 mM chenodeoxycholic acid (CDCA). ^9^ 0.17 M Cu(I) + 0.017 M Cu(II) + 0.1 M LiClO_4_ + 0.25 M TBP. ^10^ η_iodine_ = 4.3%; the amount of iodine species is equimolar to that of the copper-based couples and the same concentration of additives is present. ^11^ 0.15 M Cu(I) + 0.015 M Cu(II) + 0.1 M LiOTf + 0.25 M TBP. ^12^ η_iodine_ = 5.2%; the amount of iodine species is equimolar to that of the copper-based couples and the same concentration of additives is present. ^13^ 0.2 mM G4 + 10 mM CDCA. ^14^ 0.17 M Cu(I) + 0.017 M Cu(II) + 0.1 M LiOTf + 0.25 M TBP. ^15^ η_iodine_ = 3.4%; comparable concentrations of iodine species with respect to the copper-based couples. ^16^ 0.02 mM porphyrin-dye + 4 equivalents of CDCA. ^17^ 0.20 M Cu(I) + 0.05 M Cu(II) + 0.1 M LiTFSI + 0.5 M TBP. ^18^ 0.17 M Cu(I) + 0.017 M Cu(II) + 0.1 M LiTFSI. ^19^ η_iodine_ = 3.6%; electrolytic solution composed of 0.28 M TBP in 15/85 (v/v) mixture of valeronitrile/acetonitrile + 0.26 M *N*-methyl-*N*-butylimidazolium iodide + 0.01 M LiI + 0.017 M I_2_. ^20^ η_iodine_ = 6.1% (4.7) for Cu1, 5.6% (5.2) for Cu2, 5.8% (5.6) for Cu3; the values in parenthesis (also in the table) refer to the presence of 0.05 M guanidinium iodide. Electrolytic solution composed of 0.28 M TBP in a 15/85 (v/v) mixture of valeronitrile/acetonitrile + 0.65 M *N*-methyl-*N*-butylimidazolium iodide + 0.025 M LiI + 0.04 M I_2_.

**Table 2:** Photoelectrochemical data of solar cells filled with bipyridine copper complexes as redox mediators. ^1^

Dye	Redox Couple	V_oc_ (mV)	J_sc_ (mA cm^−2^)	FF	PCE (%)	CE	Ref
Y123 ^2^	BP1 ^3^	1070	14.15	0.687	10.0	PEDOT	[45]
Y123	BP2 ^3^	1040	15.53	0.640	10.3	PEDOT	[45]
Y123	BP2 ^4^	1070	1.50	0.71	8.01	PEDOT	[49]
Y123 ^5^	BP2 ^4^	1010	9.96	0.72	7.21	PEDOT	[49]
Y123 ^6^	BP2 ^4^	1030	10.82	0.71	7.86	PEDOT	[49]
Y123 ^2^	BP2 ^7^	1087	11.815	0.786	10.06	PEDOT	[50]
Y123	BP2 ^8^	1082	10.79	0.727	8.48	PEDOT	[50]
Y123	BP2 ^9^	1028	13.33	0.75	10.3	PEDOT	[48]
Y123 ^2^	BP3 ^10^	1080	12.392	0.781	10.42	PEDOT	[50]
Y123	BP3 ^8^	918	7.57	0.772	5.43	PEDOT	[50]
Y123 ^2^	BP4 ^7^	1010	11.851	0.783	10.18	PEDOT	[50]
Y123	BP4 ^8^	911	3.06	0.841	2.36	PEDOT	[50]
Y123	Ph2 ^11^	1060	13.61	0.692	10.3	PEDOT	[45]
D35	BP2 ^12^	1100	12.48	0.72	9.90	PEDOT	[46]
D35	BP2 ^12,13^	1010	1.63	0.74	9.90	PEDOT	[46]
D35/XY1 10:1	BP2 ^12^	1050	13.26	0.71	9.90	PEDOT	[46]
D35/XY1 10:1	BP2 ^12,13^	960	1.73	0.78	10.6	PEDOT	[46]
D35/XY1 4:1	BP2 ^12^	1030	16.19	0.68	11.3	PEDOT	[46]
D35/XY1 4:1	BP2 ^12,13^	960	2.17	0.78	13.2	PEDOT	[46]
D35/XY1 1:1	BP2 ^12^	1030	15.54	0.68	10.8	PEDOT	[46]
D35/XY1 1:1	BP2 ^12,13^	960	2.07	0.79	12.6	PEDOT	[46]
D35/XY1 1:4	BP2 ^12^	1030	14.66	0.67	10.1	PEDOT	[46]
D35/XY1 1:4	BP2 ^12,13^	970	2.03	0.78	12.3	PEDOT	[46]
D35/XY1 1:10	BP2 ^12^	1020	15.00	0.66	10.1	PEDOT	[46]
D35/XY1 1:10	BP2 ^12,13^	950	2.00	0.78	11.9	PEDOT	[46]
XY1	BP2 ^14^	1020	14.56	0.67	10.2	PEDOT	[47]
XY1	BP2 ^14,15^	960	1.94	0.78	11.8	PEDOT	[47]
XY1 ^16^	BP2 ^14^	1050	11.4	0.76	9.1	PEDOT	[47]
XY1	BP2 ^14,15^	940	1.2	0.76	8.6	PEDOT	[47]
5T ^17^	BP2 ^14^	970	9.9	0.77	7.5	PEDOT	[47]
5T	BP2 ^14,15^	870	0.96	0.78	6.5	PEDOT	[47]
XY1/5T 1:1 ^18^	BP2 ^14^	1040	11.8	0.74	9.1	PEDOT	[47]
XY1/5T 1:1 ^18^	BP2 ^14,15^	950	11.25	0.80	9.4	PEDOT	[47]
HY63	BP2 ^9^	986	13.71	0.76	10.3	PEDOT	[48]
HY63	BP2 ^9,19^	951	6.98	0.80	10.7	PEDOT	[48]
HY63	BP2 ^9,15^	884	1.37	0.78	9.5	PEDOT	[48]
HY64	BP2 ^9^	1025	15.76	0.77	12.5	PEDOT	[48]
HY64	BP2 ^9,19^	1008	7.90	0.79	12.6	PEDOT	[48]
HY64	BP2 ^9,15^	951	1.58	0.76	11.3	PEDOT	[48]
D205	BP2 ^4^	890	6.25	0.79	4.43	PEDOT	[49]
D205 ^5^	BP2 ^4^	920	5.86	0.82	4.38	PEDOT	[49]
D205 ^6^	BP2 ^4^	890	6.33	0.81	4.57	PEDOT	[49]
D205Si	BP2 ^4^	890	5.76	0.77	3.92	PEDOT	[49]
D205Si ^5^	BP2 ^4^	950	5.98	0.78	4.41	PEDOT	[49]
D205Si ^6^	BP2 ^4^	880	6.22	0.80	4.37	PEDOT	[49]
Cu4	BP1 ^20^	784	2.14	0.66	1.12	Pt	[51]
Cu4	BP5 ^21^	558	1.10	0.55	0.33	Pt	[51]
Cu5	BP1 ^20^	710	2.15	0.55	0.84	Pt	[51]
Cu5	BP6 ^22^	662	1.69	0.55	0.61	Pt	[51]
Cu5	Ph2 ^20^	812	3.09	0.72	1.82	Pt	[51]
Cu6	BP1 ^20^	679	2.21	0.64	0.97	Pt	[51]
Cu6	BP6 ^22^	655	1.88	0.52	0.64	Pt	[51]
Cu6	BP7 ^20^	681	3.44	0.75	1.76	Pt	[51]
Cu6	Ph2 ^20^	804	2.98	0.74	1.76	Pt	[51]
Cu7	BP1 ^20^	689	2.29	0.60	0.95	Pt	[51]
Cu7	BP6 ^22^	648	1.97	0.43	0.54	Pt	[51]
Cu7	BP7 ^20^	686	3.85	0.76	2.00	Pt	[51]
Cu7	Ph2 ^20^	796	2.80	0.73	1.63	Pt	[51]
Cu8	BP1 ^20^	702	2.27	0.61	0.97	Pt	[51]
Cu8	BP7 ^20^	684	4.01	0.75	2.06	Pt	[51]
Cu8	Ph2 ^20^	788	2.66	0.73	1.53	Pt	[51]

^1^ AM 1.5 simulated light source; input intensity of 100 mW cm^−2^ when not differently indicated. Data about the efficiency comparison with DSSCs filled with the same sensitizers and the I^-^/I_3_^-^ couple were not present in the works this table deals with. ^2^ with 0.4 mM CDCA. ^3^ 0.2 M Cu(I) + 0.04 M Cu(II) + 0.1 M LiTFSI + 0.6 M TBP. ^4^ 0.2 M Cu(I) + 0.04 M Cu(II) + 0.1 M LiTFSI + 0.6 M TBP. ^5^ interface modification employing CB7 molecules. ^6^ interface modification through Molecular Multicapping ^7^ 0.2 M Cu(I) + 0.05 M Cu(II) + 0.1 M LiTFSI + 0.6 M TBP. ^8^ as a zombie cell. ^9^ 0.2 M Cu(I) + 0.09 M Cu(II) + 0.1 M LiTFSI + 0.4 M 1-butyl-1*H*-benzo[*d*]imidazole. ^10^ 0.07 M Cu(I) + 0.02 M Cu(II) + 0.1 M LiTFSI + 0.6 M TBP. ^11^ as a comparison. ^12^ 0.2 M Cu(I) + 0.04 M Cu(II) + 0.1 M LiTFSI + 0.6 M tributylphosphate. ^13^ input intensity of 12 mW cm^-2^. ^14^ 0.2 Cu(I) + 0.06 M Cu(II) + 0.1 M LiTFSI + 0.6 M TBP. ^15^ input intensity of 10 mW cm^−2^. ^16^ 0.1 mM XY1 + 1 mM CDCA. ^17^ 0.1 mM D35 + 0.4 mM CDCA. ^18^ dye solution composed of equal volumes of the solutions mentioned in ^16^ and in ^17^. ^19^ input intensity of 50 mW cm^-2^. ^20^ 0.20 M Cu(I) + 0.04 M Cu(II) + 0.1 M LiPF_6_ + 0.5 M TBP. ^21^ 0.02 M Cu(I) + 0.004 M Cu(II) + 0.1 M LiPF_6_ + 0.5 M TBP. ^22^ 0.10 M Cu(I) + 0.02 M Cu(II) + 0.1 M LiPF_6_ + 0.5 M TBP.

**Table 3:** Photoelectrochemical data of solar cells filled with other types of copper complexes employed as redox mediators. ^1^

Dye	Redox Couple	V_oc_ (mV)	J_sc_ (mA cm^−2^)	FF	PCE (%)	CE	Ref
LEG4	Cu(bpye)_2_^2^	904	13.8	0.718	9.0	PEDOT	[52]
LEG4	Cu(bpye)_2_^2,3^	895	14.1	0.713	9.0	PEDOT	[52]
LEG4	Cu(bpye)_2_^2,4^	885	7.3	0.764	9.9	PEDOT	[52]
LEG4	Cu(bpye)_2_^2,5^	842	1.3	0.808	8.7	PEDOT	[52]
Y123	Cu(oxabpy) ^6^	920	9.75	0.69	6.2	PEDOT	[53]
Y123	Cu(oxabpy) ^6,7^	855	1.32	0.79	8.9	PEDOT	[53]
Y123	BP1 ^6,8^	1040	10.5	0.71	7.8	PEDOT	[53]
Y123	BP1 ^6, 7,8^	875	1.44	0.78	10.0	PEDOT	[53]
Y123	Cu(1) ^9^	689	5.7	0.77	3.1	PEDOT	[54]
Y123	Cu(1) ^9,10^	618	4.2	0.67	1.8	PEDOT	[54]
Y123	Cu(1) ^9,11^	605	2.9	0.74	1.4	PEDOT	[54]
Y123	Cu(1) ^9,12^	470	0.5	0.62	0.2	PEDOT	[54]
Y123	Cu(2) ^9^	693	10.2	0.72	4.7	PEDOT	[54]
Y123	Cu(2) ^9,13^	712	10.7	0.68	4.8	PEDOT	[54]
Y123	Cu(2) ^9,10^	784	7.4	0.76	4.5	PEDOT	[54]
Y123	Cu(2) ^9^	641	14.1	0.45	4.1	Pt	[54]
Y123	Cu(2) ^9,13^	671	13.0	0.49	4.4	Pt	[54]
Y123	Cu(2) ^9,10^	630	12.5	0.42	3.2	Pt	[54]
Y123	Cu(2) ^9,11^	686	12.3	0.47	3.9	Pt	[54]
Y123	Cu(2) ^9,14^	643	8.1	0.60	3.1	Pt	[54]
Y123	Cu(3) ^9^	792	7.9	0.75	4.3	PEDOT	[54]
Y123	Cu(3) ^9^	678	10.2	0.45	3.2	Pt	[54]
Y123	Cu(bpye)_2_^9,15^	627	13.2	0.65	5.6	PEDOT	[54]
Y123	Cu(bpye)_2_^9,15^	651	9.7	0.48	3.1	Pt	[54]

^1^ AM 1.5 simulated light source; input intensity of 100 mW cm^−2^ when not differently indicated. Data about the efficiency comparison with DSSCs filled with the same sensitizers and the I^-^/I_3_^-^ couple were not present in the works this table deals with. ^2^ 0.22 M Cu(I) + 0.05 M Cu(II) + 0.10 M LiClO_4_ + 0.20 M TBP. ^3^ under a light density of 1.0 sun. ^4^ under a light density of 0.5 sun. ^5^ under a light density of 0.1 sun. ^6^ 0.2 M Cu(I) + 0.04 M Cu(II) + 0.1 M LiTFSI + 0.6 M TBP. ^7^ input intensity of 10 mW cm^−2^. ^8^ as a reference. ^9^ 0.2 M Cu(I) + 0.04 M Cu(II) + 0.1 M LiTFSI + 0.5 M TBP. ^10^ after 24 h. ^11^ after 48 h. ^12^ after 336 h. ^13^ after 1 h. ^14^ after 72 h. ^15^ as a reference.

## Data Availability

No new data were created.

## References

[1] O’Regan B., Grätzel M. (1991). A Low-Cost, High-Efficiency Solar Cell Based on Dye-Sensitized Colloidal TiO2 Films. Nature.

[2] Nazeeruddin M.K., Klein C., Liska P., Grätzel M. (2005). Synthesis of novel ruthenium sensitizers and their application in dye-sensitized solar cells. Coord. Chem. Rev..

[3] Bredas J.-L., Durrant J.-R. (2009). Organic Photovoltaics. Acc. Chem. Res..

[4] Grätzel M. (2009). Recent advances in sensitized mesoscopic solar cells. Acc. Chem. Res..

[5] Nozik A.J., Miller J. (2010). Introduction to Solar Photon Conversion. Chem. Rev..

[6] Hagfeldt A., Boschloo G., Sun L., Kloo L., Pettersson H. (2010). Dye-Sensitized Solar Cells. Chem. Rev..

[7] Snaith H.J. (2010). Estimating the Maximum Attainable Efficiency in Dye-Sensitized Solar Cells. Adv. Funct. Mater..

[8] Caramori S., Bignozzi C.A. (2010). Electrochemistry of Functional Supramolecular Systems.

[9] Vougioukalakis G.C., Philippopoulos A.I., Stergiopoulos T., Falaras P. (2011). Contributions to the development of ruthenium-based sensitizers for dye-sensitized solar cells. Coord. Chem. Rev..

[10] Dragonetti C., Valore A., Colombo A., Roberto D., Trifiletti V., Manfredi N., Salamone M.M., Ruffo R., Abbotto A. (2012). A new thiocyanate-free cyclometallated ruthenium complex for dye-sensitized solar cells: Beneficial effects of substitution on the cyclometallated ligand. J. Organomet. Chem..

[11] Abbotto A., Coluccini C., Dell’Orto E., Manfredi N., Trifiletti V., Salamone M.M., Ruffo R., Acciarri M., Colombo A., Dragonetti C. (2012). Thiocyanate-free cyclometalated ruthenium sensitizers for solar cells based on heteroaromatic-substituted 2-aryl-pyridines. Dalton Trans..

[12] Colombo A., Dragonetti C., Valore A., Coluccini C., Manfredi N., Abbotto A. (2014). Thiocyanate-free ruthenium(II) 2,2’-bipyridyl complexes for dye-sensitized solar cells. Polyhedron.

[13] Colombo A., Dragonetti C., Magni M., Roberto D., Demartin F., Caramori S., Bignozzi C.A. (2014). Efficient Copper Mediators Based on Bulky Asymmetric Phenanthrolines for DSSCs. ACS Appl. Mater. Interfaces.

[14] Li L.-L., Diau E.W.-G. (2013). Porphyrin-sensitized solar cells. Chem. Soc. Rev..

[15] Housecroft C.E., Constable E.C. (2015). The emergence of copper(I)-based dye sensitized solar cells. Chem. Soc. Rev..

[16] Wu J., Lan Z., Lin J., Huang M., Huang Y., Fan L., Luo G. (2015). Electrolytes in Dye-Sensitized Solar Cells. Chem. Rev..

[17] Magni M., Giannuzzi R., Colombo A., Cipolla M.P., Dragonetti C., Caramori S., Carli S., Grisorio R., Suranna G.P., Bignozzi C.A. (2016). Tetracoordinated Bis-phenanthroline Copper-Complex Couple as Efficient Redox Mediators for Dye Solar Cells. Inorg. Chem..

[18] Sandroni M., Pellegrin Y., Odobel F. (2016). Heteroleptic bis-diimine copper(I) complexes for applications in solar energy conversion. C. R. Chim..

[19] Magni M., Biagini P., Colombo A., Dragonetti C., Roberto D., Valore A. (2016). Versatile copper complexes as a convenient springboard for both dyes and redox mediators in dye sensitized solar cells. Coord. Chem. Rev..

[20] Büttner A., Brauchli S.Y., Constable E.C., Housecroft C.E. (2018). Effects of introducing methoxy groups into the ancillary ligands in bis(diimine) copper(I) dyes for dye-sensitized solar cells. Inorganics.

[21] Saygili Y., Stojanovic M., Flores-Díaz N., Zakeeruddin S.M., Vlachopoulos N., Grätzel M., Hagfeldt A. (2019). Metal coordination complexes as redox mediators in regenerative dye-sensitized solar cells. Inorganics.

[22] Iftikhar H., Lund P.D., Sonai G.G., Nogueira A.F., Hashmi S.G. (2019). Progress on electrolytes development in dye-sensitized solar cells. Materials.

[23] Kavan L. (2017). Electrochemistry and dye-sensitized solar cells. Curr. Opin. Electrochem..

[24] Mathew S., Yella A., Gao P., Humphry-Baker R., Curchod B.F.E., Ashari-Astani N., Tavernelli I., Rothlisberger U., Nazeeruddin M.K., Grätzel M. (2014). Dye-sensitized solar cells with 13% efficiency achieved through the molecular engineering of porphyrin sensitizers. Nat. Chem..

[25] Yella A., Lee H.-W., Tsao H.N., Yi C., Chandiran A.K., Nazeeruddin M.K., Diau E.W.-G., Yeh C.-Y., Zakeeruddin S.M., Graetzel M. (2011). Porphyrin-Sensitized Solar Cells with Cobalt (II/III)-Based Redox Electrolyte Exceed 12% Efficiency. Science.

[26] Volz D., Zink D.M., Bocksrocker T., Friedrichs J., Nieger M., Baumann T., Lemmer U., Bräse S. (2013). Molecular Construction Kit for Tuning Solubility, Stability and Luminescence Properties: Heteroleptic MePyrPHOS-Copper Iodide-Complexes and their Application in Organic Light-Emitting Diodes. Chem. Mater..

[27] Zink D.M., Volz D., Baumann T., Mydlak M., Flügge H., Friedrichs J., Nieger M., Bräse S. (2013). Heteroleptic, Dinuclear Copper(I) complexes for Application in Organic Light-Emitting Diodes. Chem. Mater..

[28] Robertson N. (2008). CuI versus RuII: Dye-sensitized solar cells and beyond. ChemSusChem.

[29] Benesperi I., Singh R., Freitag M. (2020). Copper coordination complexes for energy-relevant applications. Energies.

[30] Lazorski M.S., Castellano F.N. (2014). Advances in the light conversion properties of Cu(I)-based photosensitizers. Polyhedron.

[31] Hattori S., Wada Y., Yanagida S., Fukuzumi S. (2005). Blue Copper Model Complexes with Distorted Tetragonal Geometry Acting as Effective Electron-Transfer Mediators in Dye-Sensitized Solar Cells. J. Am. Chem. Soc..

[32] Bai Y., Yu Q., Cai N., Wang Y., Zhang M., Wang P. (2011). High-efficiency organic dye-sensitized mesoscopic solar cells with a copper redox shuttle. Chem. Commun..

[33] Freitag M., Giordano F., Yang W., Pazoki M., Hao Y., Zietz B., Grätzel M., Hagfeldt A., Boschloo G. (2016). Copper Phenanthroline as a Fast and High-Performance Redox Mediator for Dye-Sensitized Solar Cells. J. Phys. Chem. C.

[34] Freitag M., Daniel Q., Pazoki M., Sveinbjörnsson K., Zhang J., Sun L., Hagfeldt A., Boschloo G. (2015). High-efficiency dye-sensitized solar cells with molecular copper phenanthroline as solid hole conductor. Energy Environ. Sci..

[35] Colombo A., Di Carlo G., Dragonetti C., Magni M., Orbelli Biroli A., Pizzotti M., Roberto D., Tessore F., Benazzi E., Bignozzi C.A. (2017). Coupling of Zinc Porphyrin Dyes and Copper Electrolytes: A Springboard for Novel Sustainable Dye-Sensitized Solar Cells. Inorg. Chem..

[36] Higashino T., Iiyama H., Nishimura I., Imahori H. (2020). Exploration on the Combination of Push-Pull Porphyrin Dyes and Copper(I/II) Redox Shuttles toward High-performance Dye-sensitized Solar Cells. Chem. Lett..

[37] Benazzi E., Magni M., Colombo A., Dragonetti C., Caramori S., Bignozzi C.A., Grisorio R., Suranna G.P., Cipolla M.P., Manca M. (2018). Bis(1,10-phenanthroline) copper complexes with tailored molecular architecture: From electrochemical features to application as redox mediators in dye-sensitized solar cells. Electrochim. Acta.

[38] Higashino T., Iiyama H., Nimura S., Kurumisawa Y., Imahori H. (2020). Effect of Ligand Structures of Copper Redox Shuttles on Photovoltaic Performance of Dye-Sensitized Solar Cells. Inorg. Chem..

[39] Dragonetti C., Magni M., Colombo A., Melchiorre F., Biagini P., Roberto D. (2018). Coupling of a Copper Dye with a Copper Electrolyte: A Fascinating Springboard for Sustainable Dye-Sensitized Solar Cells. ACS Appl. Energy Mater..

[40] Dragonetti C., Magni M., Colombo A., Fagnani F., Roberto F., Melchiorre F., Biagini P., Fantacci S. (2019). Towards efficient sustainable full-copper dye-sensitized solar cells. Dalton Trans..

[41] Colombo A., Dragonetti C., Fagnani F., Roberto D., Melchiorre F., Biagini P. (2019). Improving the efficiency of copper-dye-sensitized solar cells by manipulating the electrolyte solution. Dalton Trans..

[42] Kavan L., Saygili Y., Freitag M., Zakeeruddin S.M., Hagfeldt A., Grätzel M. (2017). Electrochemical Properties of Cu(II/I)-Based Redox Mediators for Dye-Sensitized Solar Cells. Electrochim. Acta.

[43] Kavan L., Krysova H., Janda P., Tarabkova H., Saygili Y., Freitag M., Zakeeruddin S.M., Hagfeldt A., Grätzel M. (2017). Novel highly active Pt/graphene catalyst for cathodes of Cu(II/I)-mediated dye-sensitized solar cells. Electrochim. Acta.

[44] Ferdovsi P., Saygili Y., Zakeeruddin S.M., Mokhtari J., Grätzel M., Hagfeldt A., Kavan L. (2018). Alternative bases to 4-*tert*-butylpyridine for dye-sensitized solar cells employing copper redox mediator. Electrochim. Acta.

[45] Saygili Y., Söderberg M., Pellet N., Giordano F., Cao Y., Muñoz-García A.B., Zakeeruddin S.M., Vlachopoulos N., Pavone M., Boschloo G. (2016). Copper Bipyridyl Redox Mediators for Dye-Sensitized Solar Cells with High Photovoltage. J. Am. Chem. Soc..

[46] Freitag M., Teuscher J., Saygili Y., Zhang X., Giordano F., Liska P., Hua J., Zakeeruddin S.M., Moser J.-E., Grätzel M. (2017). Dye-sensitized solar cells for efficient power generation under ambient lighting. Nature Photonics.

[47] Tanaka E., Michaels H., Freitag M., Robertson N. (2020). Synergy of co-sensitizers in a copper bipyridyl redox system for efficient and cost-effective dye-sensitized solar cells in solar and ambient light. J. Mater. Chem. A.

[48] Jiang H., Ren Y., Zhang W., Wu Y., Socie E.C., Irving Carlsen B., Moser J.-E., Tian H., Zakeeruddin M.S., Zhu W.-H. (2020). Phenanthrene-Fused-Quinoxaline as a Key Building Block for Highly Efficient and Stable Sensitizers in Copper-Electrolyte-Based Dye-Sensitized Solar Cells. Angew. Chem. Int. Ed..

[49] Glinka A., Gierszewski M., Gierczyk B., Burdziński G., Michaels H., Freitag M., Ziółek M. (2020). Interface Modification and Exceptionally Fast Regeneration in Copper Mediated Solar Cells Sensitized with Indoline Dyes. J. Phys. Chem. C.

[50] Saygili Y., Stojanovic M., Kim H.-S., Teuscher J., Scopelliti R., Freitag M., Zakeeruddin S.M., Moser J.-E., Grätzel M., Hagfeldt A. (2020). Liquid State and Zombie Dye Sensitized Solar Cells with Copper Bipyridine Complexes Functionalized with Alkoxy Groups. J. Phys. Chem. C.

[51] Karpacheva M., Malzner F.J., Wobill C., Büttner A., Constable E.C., Housecroft C.E. (2018). Cuprophilia: Dye-sensitized solar cells with copper(I) dyes and copper(I)/(II) redox shuttles. Dyes Pigment..

[52] Cong J., Kinschel D., Daniel Q., Safdari M., Gabrielsson E., Chen H., Svensson P.H., Sun L., Kloo L. (2016). Bis(1,1-bis(2-pyridyl)ethane)copper(I/II) as an efficient redox couple for liquid dye-sensitized solar cells. J. Mater. Chem. A.

[53] Michaels H., Benesperi I., Edvinsson T., Muñoz-Garcia A.B., Pavone M., Boschloo G., Freitag M. (2018). Copper Complexes with Tetradentate Ligands for Enhanced Charge Transport in Dye-Sensitized Solar Cells. Inorganics.

[54] Rodrigues R.R., Lee J.M., Taylor N.S., Cheema H., Chen L., Fortenberry R.C., Delcamp J.H., Jurss J.W. (2020). Copper-based redox shuttles supported by preorganized tetradentate ligands for dye-sensitized solar cells. Dalton Trans..

